# Immediate Ocular Changes After Light-Emitting Diode Displays Exposure—A Preliminary Study

**DOI:** 10.3389/fmed.2022.848794

**Published:** 2022-04-04

**Authors:** Chia-Chen Lin, Jia-Horung Hung, Yi-Hsun Huang

**Affiliations:** ^1^Department of Ophthalmology, College of Medicine, National Cheng Kung University Hospital, National Cheng Kung University, Tainan, Taiwan; ^2^Institute of Clinical Medicine, College of Medicine, National Cheng Kung University, Tainan, Taiwan

**Keywords:** computer vision syndrome (CVS), dry eye disease (DED), light-emitting diodes (LED), lipid layer thickness (LLT), video display terminals (VDT)

## Abstract

**Purpose:**

Computer vision syndrome (CVS) is one of the most frequently encountered problems among video display terminals (VDT) users, but little is known regarding the short-term effect after exposure to light-emitting diodes (LED). The purpose of this study was to determine if short-term exposure to LED leads to changes in corrected distance visual acuity (CDVA), lipid layer thickness (LLT), blink rates, partial blink ratio, and computer vision syndrome questionnaire (CVS-Q) score.

**Design:**

Prospective, cross-sectional study.

**Methods:**

In this study, participants were recruited at the National Cheng-Kung University Hospital, a tertiary referral center in southern Taiwan, for examination. Participants were asked to complete the CVS-Q and undergo a series of ocular examinations, including CDVA, LLT, blink rates and partial blink ratio before and after watching an LED display for 15 min. Main Outcome Measures were changes in CDVA, LLT, blink rates, partial blink ratio, and CVS-Q measurements.

**Results:**

In total, 120 eyes from 60 participants (mean age: 35.7 ± 9.4 years) were included; 31 participants were men (51.7%), and 29 were women (48.3%). The CDVA, LLT, blink rates, and partial blink ratio did not change after watching the LED display. The CVS-Q score significantly improved after short-term LED exposure (*P* < 0.001). A subgroup analysis of subjects with a baseline LLT of <60 nm or ≥60 nm determined that LLT significantly decreased in individuals with a baseline LLT of ≥60 nm (*P* = 0.016).

**Conclusion:**

Short-term use of LED displays reduced LLT in individuals with a baseline LLT of ≥60 nm, despite the visual symptoms of CVS improved subjectively. Therefore, digital device users should be aware of the potential negative effects of LED exposure on the eyes.

## Introduction

Video display terminals (VDTs) are ubiquitous, and engagement in digital screens has grown substantially across all age groups worldwide (1). During the coronavirus disease 2019 (COVID-19) pandemic, lockdowns and increasing demand for digital learning and working have led to more frequent and sustained VDT use (2, 3). Prolonged exposure to VDTs is associated with the development of various health problems, including psychosocial issues, venous thromboembolism, fatigue, and visual complaints (4–6).

Computer vision syndrome (CVS) is one of the most frequently encountered problems among VDT users (7). CVS comprises several visual and musculoskeletal symptoms resulting from VDT use, such as eye strain, dryness and burning sensation of the eye, blurred vision, and neck and shoulder pain (8–10). In previous studies, high frequency of dry eye and VDT-associated ocular symptoms including soreness, itchiness, dryness, foreign body sensations and pain was found after long-term VDT use (11, 12). A major alteration is the development of dry eye disease (DED), occurring in 60% of those with CVS (13). Dry eye symptoms, corneal erosions, short tear-film breakup time (BUT), low tear meniscus height, and meibomian gland dysfunction (MGD) are all DED presentations encountered by VDT users (14–17). The continuous use of VDTs is an established risk factor for CVS and DED (18). However, it is unclear whether transient exposure to VDTs leads to ocular surface changes, especially regarding lipid layer thickness (LLT).

This study investigated if the short-term use of light-emitting diode (LED) displays (one type of VDT) changed the ocular parameters associated with CVS or DED, including corrected distance visual acuity (CDVA) (19–21), the CVS-Questionnaire (CVS-Q) score, the blink rate, the partial blink ratio, and LLT in healthy, working age population. The CVS-Q helps quantify associated ocular discomforts and is a reliable tool to assess VDT-related symptoms (22). The blink rates, partial blink ratio and the LLT all contribute to tearfilm stability as well as ocular surface homeostasis (23). To our knowledge, this is the first study to focus on the immediate effects of LEDs on the eye.

## Methods

### Study Design

This prospective, open-label clinical study was conducted in the Ophthalmology Department of the National Cheng Kung University Hospital (NCKUH), Tainan, Taiwan. The study was approved by the Institutional Review Board of NCKUH (IRB No.: A-ER-108-489) and followed the tenets of the Declaration of Helsinki. Written informed consent was obtained from all the participants.

### Sample Size

Sample size estimation was performed using G^*^Power software version 3.1.9.2 [Faul, Erdfelder, (24)]. Statistical tests built on a presumed dataset of paired 26 cases pre- and post- LED exposure will have 80+% power to successfully detect the difference with effect size > 0.5 for each group. Thus, we aimed to recruit 30 patients for each group to prevent dropout or no-show, and there comes the final estimation of 60 patients for this study.

### Eligibility

The inclusion criteria were generally healthy working age individuals not under systemic or ocular medication, aged between 20 and 65 years who were willing to participate in the study. The exclusion criteria were ages below 20 or over 65 years, a history of using any systemic or ocular medication for the past 3 months, and a history of ocular diseases or previous ocular surgery. Amblyopia with a CDVA score of <0.1 on the Landolt C chart in either eye or a CDVA difference of >0.2 between the eyes were also excluded to better represent general population. A baseline IOP higher than 25 mmHg was also excluded to avoid potential participants with undiagnosed ocular hypertension or glaucoma. Any eye with ocular pathology, including macular edema, scarring, epiretinal membrane, hyperreflectivity, or areas of abnormal hypo- or hyper-autofluorescence identified on fundus examination by optical coherence tomography (OCT) or fundus autofluorescence (FAF) imaging were excluded.

### Participant Recruitment

Participants were recruited through printed and online advertisement at various sites, including university campuses, local communities, and large companies in Tainan City, Taiwan. The advertisement described the nature of the study and our eligibility criteria. Before the examination day, volunteers were contacted via phone or email to confirm their age and medical history as initial screening.

### Study Procedure

All participants completed the following study procedures in the same day. Participants were instructed to fill out a basic information form, including their name, sex, age, and contact information. They were also informed to avoid wearing contact lenses for 2 days before the test. We considered 2 days sufficient to eliminate the influence of contact lenses on the ocular surface based on a previous research, showing that tear dynamics are not significantly altered after removal of contact lenses (25). For the baseline test, the participants were asked to complete the CVS-Q to assess their CVS (22). The questionnaire evaluates frequency and intensity of symptoms related to CVS using a single rating scale that fits the Rasch rating scale (26). The sensitvity and specificity are over 70% with good test-retest repeatability. For application of the CVS-Q to the current study, the questionnaire was translated to Chinese for the study population (Supplementary Material). Next, a series of ocular examinations were performed in the following order, with ~5 s of time lapse in between—CDVA, IOP, LLT and blink patterns, an OCT scan of the macula, and fundus FAF imaging. The CDVA was obtained with correction of the refraction errors measured by an autorefractor (Topcon Corporation, Tokyo, Japan). IOP was measured with non-contact air puff tonometers (Topcon Corporation, Tokyo, Japan). The LLT, blink rates and partial blink ratio were measured using the LipiView II Ocular Surface Interferometer following the standard protocol (TearScience, Morrisville, NC, USA). Central retinal thickness (CRT) was measured with macular OCT scan. After the examination, the participants watched a short movie on an LED screen (InnoLux, Taiwan) for 15 min in a bright room. The illuminance was 600 lux, measured with a handheld energy meter (Ophir Optronics, Israel). The screen is a 55 inch monitor. The resolution is 960 × 540 pixels and the luminance is 600 nits (Supplementary Material). The viewing distance was ~1.5 m. After the movie ended, the participants were instructed to complete the CVS-Q, which took ~1 min, and undergo the CDVA measurement followed by LLT and blink patterns tests again. Since this study investigates the immediate change after LED exposure, during the post-test, we instructed the subjects to focus on the symptoms after 15 min of screen watching when completing the post-test CVS-Q. The baseline CDVA, IOP, OCT, and FAF images of each participant were reviewed by ophthalmologists to screen for clinically significant ocular pathology.

### Statistical Analyses

Statistical analyses were performed using R software version 4.1.0 (R Core Team, Vienna, Austria) and SAS Enterprise Guide (version 9.4; SAS Institute Inc., Cary, NC, USA). Shapiro–Wilk method was used for test of normality. Continuous variables were presented as the means and standard deviations for parametric data, and median and ranges for non-parametric data. CDVA was obtained in Snellen values and converted to the logarithm of the minimum angle of resolution (logMAR) for analysis. The generalized estimating equation (GEE) linear models, which takes into account correlations between fellow eyes (27), were used to compare the CDVA, CVS-Q score, LLT, blink rate, and the partial blink ratio before and after watching the LED display. The fitness of GEE is assessed according to quasi-likelihood information criterion (28). Statistical significance was set at *P* < 0.05 with two-tailed test.

### Subgroup Analysis

The LLT, blink rates, partial blink ratios and CVS-Q score were further analyzed between participants with high (≥60) and low (<60) baseline LLTs. The cutoff value was 60 nm because previous studies reported that a thin LLT (<60 nm) significantly correlated with DED (23, 29). Korb et al. investigated how blinking improved LLT and found that subjects with baseline LLT values < or = 60 nm experienced a mean increase of 19 nm of LLT after forceful blinking (30). Another study also revealed LLT values < or = 60 nm was correlated with lower tear break-up time and Schirmer's test score (31).

## Results

### Clinical Characteristics

This study examined 120 eligible eyes from 60 study subjects; 31 patients were male, and 29 were female. Table 1 presents the baseline characteristics. The mean age was 35.7 ± 9.4 years, the mean CDVA was 0.05 ± 0.10 (logMAR), and the mean IOP was 17.6 ± 3.1 mm Hg. The average baseline CVS-Q score was 4.9 ± 5.1, and the average baseline LLT was 63.2 ± 21.2 nm.

**Table 1:** Clinical characteristics of the study population.

**Characteristics**	**Total**
	***n* = 60**
**Age (years)**	
Mean ± SD	35.7 ± 9.4
Median	35
Range	20–61
20–29	15 (25.0)
30–39	30 (50.0)
40–49	8 (13.3)
50–59	5 (8.3)
60–69	2 (3.3)
**Male (** ***n*** **, %)**	31 (51.7%)
Mean ± SD (years)	34.5 ± 7.3
Median	35
Range	20–54
**Female (** ***n*** **, %)**	29 (48.3%)
Mean ± SD (years)	37.1 ± 11.3
Median	35
Range	22–61
**Baseline visual acuity**	
**Landolt C chart**	
Median	1.00
Range	0.30–1.00
**LogMAR**	
Mean ± SD	0.05 ± 0.10
Range	0.00–0.52
(Landolt C equivalent)	0.88
**Baseline IOP (mm Hg)**	
Mean ± SD	17.6 ± 3.1
Median	18.0
Range	10–25
**Baseline CRT (μm)**	
Mean ± SD	237.3 ± 18.9
Median	238
Range	168–286

*SD, standard deviation; LogMAR, logarithm of the Minimum Angle of Resolution; IOP, intraocular pressure; CRT, central retinal thickness*.

### Ocular Parameters Before and After LED Exposure

Table 2 presents the ocular parameters before and after watching the LED display. The CVS-Q score was significantly lower (*P* < 0.001) after VDT use. CDVA did not differ. The LLT decreased from 63.2 ± 21.2 nm to 61.9 ± 19.8 nm and the blink rates reduced from 5.0 ± 3.8/min to 4.6 ± 3.5/min but did not reach statistical significance. The partial blink ratio increased from 68.7% before to 72.0% after LED exposure but was statistically insignificant.

**Table 2:** The ocular parameters before and after watching a movie for 15 min.

**Characteristics**	**Before *n* = 120**	**After *n* = 120**	**Difference (95% CI)**	***P*-value**
**Visual acuity**				
**Landolt C chart**				NA
Median	1.0	1.0		
Range	0.30–1.0	0.40–1.0		
**LogMAR**				0.303
Mean ± SD	0.05 ± 0.10	0.05 ± 0.08	−0.009 (−0.026, 0.008)	
Range	0.00–0.52	0.00–0.40		
(Landolt C equivalent)	0.88	0.90		
**LLT (nm)**				0.530
Mean ± SD	63.2 ± 21.2	61.9 ± 19.8	−1.250 (−5.155, 2.655)	
Median	60.0	61.0		
Range	28–100	29–100		
**Blink rate (/min)**				0.280
Mean ± SD	5.0 ± 3.8	4.6 ± 3.5	−0.425 (−1.196, 0.346)	
Median	4.0	4.0		
Range	0–21	0–25		
**Partial blink ratio (%)**				0.189
Mean ± SD	68.7 ± 37.3	72.0 ± 36.5	3.706 (−2.544, 9.956)	
Median	83.3	100.0		
Range	0–100	0–100		
**CVS-Q score**				<0.001
Mean ± SD	4.9 ± 5.1	2.2 ± 2.6	−2.767 (−3.824, −1.711)	
Median	3	1		
Range	0–25	0–14		

*CI, confidence of interval; LogMAR, logarithm of the Minimum Angle of Resolution; SD, standard deviation; IOP, intraocular pressure; LLT, lipid layer thickness; CVS, computer vision syndrome*.

### Subgroup Analysis

The baseline characteristics of the two subgroups were compared (Table 3). Age, sex ratio, CDVA, and IOP did not differ between the low and high LLT groups. The CVS-Q score of the high baseline LLT group was lower than the low baseline LLT group (high: 3.9 ± 3.2 vs. low: 6.0 ± 6.2), but the difference was statistically insignificant. However, the LLT of the participants in the high baseline LLT group significantly decreased after watching the LED display (*P* = 0.016; Table 4).

**Table 3:** Participants characteristics based on their baseline lipid layer thickness.

**Characteristics**	**Baseline LLT <60 *n* = 29**	**Baseline LLT ≥60 *n* = 31**	***P*-value**
**Age (years)**			0.477
Mean ± SD	36.8 ± 10.8	34.7 ± 8.1	
Median	35	35	
Range	20–61	20–55	
**Male (** ***n*** **, %)**	17 (58.6%)	14 (45.2%)	0.297
**Baseline visual acuity**			
**Landolt C chart**			
Median	1.00	1.00	
Range	0.40–1.00	0.30–1.00	
**LogMAR**			0.289
Mean ± SD	0.06 ± 0.09	0.05 ± 0.10	
Range	0.00–0.40	0.00–0.52	
(Landolt C equivalent)	0.87	0.89	
**Baseline IOP (mm Hg)**			0.748
Mean ± SD	17.8 ± 3.0	17.5 ± 3.2	
Median	18.0	17.5	
Range	11–25	10–25	
**Blink rate (/min)**			0.398
Mean ± SD	5.5 ± 3.7	4.7 ± 3.3	
Median	5.0	3.5	
Range	0.5–14	1–14.5	
**Partial blink ratio (%)**			0.165
Mean ± SD	68.8 ± 32.0	68.5 ± 32.3	
Median	75.0	78.6	
Range	0–100	0–100	
**CVS-Q score**			0.205
Mean ± SD	6.0 ± 6.2	3.9 ± 3.6	
Median	4	3	
Range	0–25	0–13	
**LLT (nm)**			<0.001
Mean ± SD	46.6 ± 7.9	78.7 ± 13.3	
Median	47.5	78.5	
Range	32.5–58.5	60–100	

*LLT, lipid layer thickness; SD, standard deviation; IOP, intraocular pressure; CVS, computer vision syndrome*.

**Table 4:** Changes after watching an LED display based on the participants' baseline lipid layer thickness.

**Characteristics**	**Before**	**After**	**Difference (95% CI)**	***P*-value**
**Baseline LLT** **<** **60 (*****n*** **=** **29)**
**LLT (nm)**				0.084
Mean ± SD	46.6 ± 7.9	51.3 ± 14.7	4.724 (−0.355, 9.803)	
Median	47.5	49.0		
Range	32.5–58.5	32.5–96		
**Blink rate (/min)**				0.067
Mean ± SD	5.5 ± 3.7	4.3 ± 2.2	−1.172 (−2.175, 0.170)	
Median	5.0	4.0		
Range	0–14	0–11		
**Partial blink ratio (%)**				0.367
Mean ± SD	68.8 ± 32.0	73.1 ± 34.9	2.524 (−6.491, 11.540)	
Median	75.0	88.0		
Range	0–100	0–100		
**CVS-Q score**				<0.001
Mean ± SD	6.0 ± 6.2	2.0 ± 2.9	−4.000 (−5.712, −2.288)	
Median	4	1		
Range	0–25	0–14		
**Baseline LLT** **≥60 (*****n*** **=** **31)**				
**LLT (nm)**				0.016
Mean ± SD	78.7 ± 13.3	71.8 ± 16.6	−6.839 (−11.990, −1.687)	
Median	78.5	67.5		
Range	60.0–100.0	46.0–100.0		
**Blink rate (/min)**				0.575
Mean ± SD	4.6 ± 3.2	4.9 ± 3.9	0.443 (−0.429, 1.314)	
Median	3.5	4.0		
Range	1–14.5	0–18.5		
**Partial blink ratio (%)**				0.650
Mean ± SD	70.5 ± 37.0	78.7 ± 31.4	8.929 (−1.204, 19.062)	
Median	88.9	100.0		
Range	0–100	0–100		
**CVS-Q score**				0.010
Mean ± SD	3.9 ± 3.6	2.3 ± 2.3	−1.613 (−2.739, −0.487)	
Median	3	1		
Range	0–13	0–9		

*CI, Confidence interval; LLT, lipid layer thickness; SD, standard deviation; CVS, computer vision syndrome*.

## Discussion

This study investigated the effects of short-term exposure to LED displays on ocular parameters. Notably, we found that LLT significantly decreased in subjects with a higher baseline LLT (≥60 nm). Previous studies revealed that VDT use was associated with shorter tear-film BUT (13, 32), and there was strong correlation between LLT and BUT (31). The BUT was not performed in this study because it requires instilling topical fluorescein into the eyes, which we considered invasive enough to interfere with LLT measurement and CVS-Q assessment before and after the LED exposure. Animal models have also demonstrated abnormal lacrimal gland function with chronic exposure to VDTs (33, 34). Accordingly, our finding regarding the significant LLT decrease after short-term LED exposure helps to complete the pathogenesis of VDT-associated dry eye (18).

The results showed that CDVA did not change significantly after short-term VDT exposure. Our interest in visual acuity stems from previous literature suggesting that dry eye and computer vision syndrome are associated with vision deterioration. They cause irregularity of the ocular surface and reduce the quality of optical image (19). Worse visual acuity was associated with worse Ocular Surface Disease Index score (20), and blurred vision is a major symptom encountered by individuals with CVS or dry eye (21). Our results implicated that short-term VDT exposure may not result in significant visual disturbance. This may be explained by the lack of significant change in other ocular surface parameters including LLT, blink rates, or partial blink ratio.

Several studies have demonstrated a reduction in the blink rate during VDT use to ~33% (21, 35–37). Incomplete blinking is equally implicated regarding CVS development (38, 39). Regarding blinking, decreased frequency and poor movement are associated with increased tear evaporation, poor tear film stability maintenance, and decreased lipid excretion from the meibomian glands, all of which contribute to DED. However, in our study, the blink rates and the partial blink ratio did not change after 15 min of LED exposure. Several factors may lead to this result. First, we focused on short-term changes after VDT use and did not measure the blink rates or the partial blink ratio during VDT use. After watching VDT, the blinking patterns and frequency were measured under relaxed conditions as that in baseline measurements, which may explain the similar blinking pattern before and after test. Second, the VDT use duration in our study was relatively short compared to previous studies. Cumulative time positively correlates with CVS and DED development (40, 41). Therefore, short-term exposure may not alter the blinking frequency and pattern. Third, our participants did not have a known history of DED. Previous studies found significantly reduced blinking after initiating VDT use, which remained low after 30 min in patients with moderately dry eyes (36). Moreover, reduced blinking is exacerbated in individuals with preexisting DED (39). Taken together, the better baseline condition of our participants may have contributed to the unchanged blinking pattern in this study.

Our results showed that LLT decreased significantly after LED exposure in individuals with higher baseline LLT. We suppose the following potential underlying mechanisms. First, as discussed above, previous literature showed decreased blinking during VDT use. Lid movements affect the composition and stability of tear film (42). In addition, blink patterns are associated with the development of MGD, which potentially may lead to decrease in LLT (43). Although blink rates and partial blink ratio did not change significantly in our study after LED exposure, as discussed in the previous paragraph, we did not measure these parameters during VDT use. Second, animal study has shown that LED-derived blue light overexposure can induce ocular surface inflammation and dry eye (44). Although LLT was not analyzed in the study, the results revealed decreased tear breakup time with exposure to LED light. Since BUT and LLT could be correlated, it was possible that LLT be affected as well.

On the other hand, LLT did not change in individuals with a lower baseline LLT (<60 nm). We surmise that since the baseline LLT was low, further significant damage was difficult to ascertain after brief exposure to VDTs. Korb et al. investigated how blinking improved LLT and found that the magnitude of the increase positively correlated with the baseline LLT values (45). Likewise, individuals with a lower baseline LLT could have a smaller magnitude of change, which was insignificant. Finally, the CVS-Q was the questionnaire of choice in this study because it is a reliable and validated questionnaire with high sensitivity, specificity, and good test-retest repeatability developed specifically for VDT users (22, 46, 47). Intriguingly, the post-test CVS-Q score was better in our study. The discrepancy between the change in visual symptoms and the change in LLT implies that objective damage to the ocular surface can occur before people are subjectively aware of any discomfort.

Although we found that LLT changed after short-term LED exposure, this study has some limitations. First, the sample size was small and the age distribution was uneven, further large-scale studies are required to confirm our preliminary results. Second, in order to streamline the study procedures to evaluate the immediate change in ocular parameters after LED exposure, the assessment of meibomian gland expression and quality was not included in our study. However, LLT values are affected in individuals with MGD. Studies focusing on patients with MGD should be conducted in the future. Third, we only observed the immediate changes of ocular surface parameters after LED exposure. Further investigations with prolonged observation time are needed to determine the duration of the effects. Fourth, the study design was open-label, with both the investigators and participants aware of the LED screen exposure. Thus, the outcomes may be subject to bias. However, the prospective design strengthens this study. Additionally, the study was performed under uniform conditions at the same day in NCKUH, which means that the environment was well-controlled. We also conducted a thorough baseline ophthalmic exam for every participant to exclude ocular diseases that could interfere with the results.

In conclusion, our results demonstrate that short-term exposure to LED displays significantly reduced LLT in individuals with a higher baseline LLT (≥60 nm). CVS symptoms did not progress after short-term VDTs use. However, there might be long-term effects regarding decreased LLT, further contributing to DED. In the era of COVID-19, while there is the intensive use of digital devices, the public should be aware that engagement with LED displays could have a negative, non-symptomatic effect on the eyes, even with only short-term exposure. Future studies are required to evaluate whether the effects are cumulative.

## Data Availability Statement

The original contributions presented in the study are included in the article/Supplementary Material, further inquiries can be directed to the corresponding authors.

## Ethics Statement

The studies involving human participants were reviewed and approved by Institutional Review Board of National Cheng Kung University Hospital (IRB No.: A-ER-108-489). The patients/participants provided their written informed consent to participate in this study.

## Author Contributions

C-CL, J-HH, and Y-HH involved in design and conduct of study, participated in management, interpretation of the data, and preparation of the manuscript. Collection and management of the data were done by C-CL, J-HH, and Y-HH. J-HH and Y-HH participated in review and approval of the manuscript. All authors contributed to the article and approved the submitted version.

## Funding

This study was supported by grants from National Cheng Kung University Hospital, Tainan, Taiwan (NCKUH-11102004), and the Ministry of Science and Technology (MOST 110-2314-B-006-086-MY3) to J-HH. The sponsor or funding organization had no role in the design or conduct of this research.

## Conflict of Interest

The authors declare that the research was conducted in the absence of any commercial or financial relationships that could be construed as a potential conflict of interest.

## Publisher's Note

All claims expressed in this article are solely those of the authors and do not necessarily represent those of their affiliated organizations, or those of the publisher, the editors and the reviewers. Any product that may be evaluated in this article, or claim that may be made by its manufacturer, is not guaranteed or endorsed by the publisher.

## Supplementary Material

Supplementary Figure 1 The LED screen used in the current study.
