# Supplementary material for: Immediate Ocular Changes After Light-Emitting Diode Displays Exposure—A Preliminary Study
Source: Front Med (Lausanne). 2022 Apr 4;9:848794. doi: 10.3389/fmed.2022.848794 (PMC9015093; doi:10.3389/fmed.2022.848794)
Supplement: Supplementary file 1 [file Data_Sheet_1.pdf]

LED顯示器觀賞前

A. 平時視覺症狀 – 請回答您是否出現以下症狀，並勾選相對應的頻率和強度。

B. 解說如下：

1. 頻率：無 = 過去一週內皆無此項症狀
   有時 = 過去一週內曾感受到這項不適，但不超過一半的時間
   經常或總是=過去一週內曾感受到這項不適，且超過一半的時間
2. 強度：*若您無此項症狀，於『頻率』處勾選『無』，則不必勾選強度。*

	1. 頻率			2. 強度	
	無	有時	經常或總是	中等	強烈
**1. 眼睛有灼熱感**					
**2. 眼睛感覺癢**					
**3. 眼睛會有進沙的感覺（異物感）**					
**4. 眼睛流淚**					
**5. 一直眨眼睛**					
**6. 注意到眼睛紅紅的**					
**7. 眼睛會有疼痛不適感**					
**8. 眼皮感覺重重的**					
**9. 眼睛乾澀**					
**10. 眼睛看東西會覺得模糊不清**					
**11. 眼睛出現複視的情形（一個東西看成兩個或出現疊影）**					
**12. 看近的東西覺得難以對焦**					
**13. 眼睛對光線敏感（畏光）**					
**14. 看東西時旁邊出現彩色的光圈**					
**15. 感覺視力逐漸變差**					
**16. 頭痛**					

LED顯示器觀賞後

A. 請回答您此次觀賞 LED 顯示器時或觀賞後是否出現以下症狀，並勾選相對應的頻率和強度。

B. 解說如下：
   1. 頻率：無 = 無論是觀賞時或觀賞後皆無此項症狀
      有時 = 觀賞時或觀賞後會感受到這項不適，但不超過一半的時間
      經常或總是=觀賞時或觀賞後感受到這項不適，且超過一半的時間
   2. 強度：*若您無此項症狀，於『頻率』處勾選『無』，則不必勾選強度。*

	1. 頻率			2. 強度	
	無	有時	經常或總是	中等	強烈
**1. 眼睛有灼熱感**					
**2. 眼睛感覺癢**					
**3. 眼睛會有進沙的感覺（異物感）**					
**4. 眼睛流淚**					
**5. 一直眨眼睛**					
**6. 注意到眼睛紅紅的**					
**7. 眼睛會有疼痛不適感**					
**8. 眼皮感覺重重的**					
**9. 眼睛乾澀**					
**10. 眼睛看東西會覺得模糊不清**					
**11. 眼睛出現複視的情形（一個東西看成兩個或出現疊影）**					
**12. 看近的東西覺得難以對焦**					
**13. 眼睛對光線敏感（畏光）**					
**14. 看東西時旁邊出現彩色的光圈**					
**15. 感覺視力逐漸變差**					
**16. 頭痛**					
