# Supplementary figures and images for: Immediate Ocular Changes After Light-Emitting Diode Displays Exposure—A Preliminary Study
Source: Front Med (Lausanne). 2022 Apr 4;9:848794. doi: 10.3389/fmed.2022.848794 (PMC9015093; doi:10.3389/fmed.2022.848794)

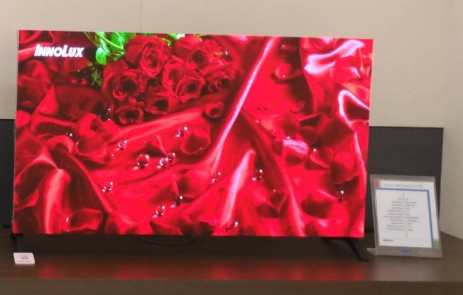

Supplement: Supplementary Figure 1 — The LED screen used in the current study. [file Image_1.jpg]
